# Inhibition of ASIC1a-Mediated ERS Improves the Activation of HSCs and Copper Transport Under Copper Load

**DOI:** 10.3389/fphar.2021.653272

**Published:** 2021-05-31

**Authors:** Lingjin Kong, Huiping Huang, Shaohua Luan, Hui Liu, Manping Ye, Fanrong Wu

**Affiliations:** Inflammation and Immune Mediated Diseases Laboratory of Anhui Province, School of Pharmacy, Anhui Institute of Innovative Drugs, Anhui Medical University, Hefei, China

**Keywords:** hepatolenticular degeneration, liver fibrosis, ASIC1a, endoplasmic reticulum stress, P38MAPK signaling pathways

## Abstract

Hepatolenticular degeneration (HLD) is an autosomal recessive genetic disease caused by the toxic accumulation of copper in the liver. Excessive copper will disrupt the redox balance in cells and tissues, causing ischemia, hypoxia, and inflammation. Acid-sensitive ion channel 1a is a cationic channel activated by extracellular acid and allowing Ca^2+^ and Na^+^ to flow into cells. Its expression appears in inflammation, arthritis, fibrotic tissue, and damaged environment, but its role in hepatolenticular degeneration has not been studied. This study established a Wistar rat model of high copper accumulation and used CuSO_4_ to induce the activation of HSC-T6 in an *in vitro* experiment. *In vivo*, Wistar rats were examined to determine the serum copper concentration, serum ALT and AST activities, and liver copper accumulation, and liver tissue HE staining and immunohistochemical analyses were conducted. The expression of ASIC1a, α-SMA, Collagen-Ι, GRP78, XBP1, ATP7B, and CCS were detected. Besides, immunofluorescence technology can detect the expression of the phosphorylated protein *in vitro*. It is suggested that ASIC1a is involved in the quality control of the endoplasmic reticulum, which degrades mutant ATP7B and increases the accumulation of copper. After blocking or silencing the expression of ASIC1a, ELISA can detect the level of inflammatory factors, the expression of endoplasmic reticulum stress-related factors, and ATP7B was improved in a higher copper environment reduction of copper deposition was observed in liver Timm’s staining. Collectively, we conclude that ASIC1a is involved in the HSC activation induced by copper accumulation and promotes the occurrence of hepatolenticular fibrosis.

## Introduction

Hepatolenticular degeneration (HLD) is an autosomal recessive copper metabolism disorder caused by mutations in the ATP7B gene (Lutsenko et al., 2007; Schilsky, 2014). The clinical manifestation of HLD is the accumulation of copper in multiple organs, including the liver, cornea, and nervous system; although the rate, location, and degree of copper deposition in the body are different and the clinical symptoms are complex and diverse, all patients have different degrees of chronic liver damage (Ala et al., 2007; Xu et al., 2013; Mensing et al., 2018). The excretion of copper ions enters the biliary tract, and free copper accumulates in the liver, causing inflammation and acidification of the tissue, which activates hepatic stellate cells. The activated hepatic stellate cells are transformed into muscle fiber-like cells (MFB), accompanied by the synthesis and secretion of α-smooth muscle actin (α-SMA) and excess extracellular matrix (extracellμLarmatrix, ECM). ECM is unbalanced degradation and deposition in the liver, making liver fibrosis the main pathological change in the liver of HLD patients (Sun and Kisseleva, 2015; Gerosa et al., 2019). A previous study by our group found that the expression level of ASIC1a was increased in HSC-T6 cells activated by acid, and its increased expression occurred downstream of the phosphorylation of components of the MAPK signalling pathway, which transmits stimulus signals. The accumulation of copper in hepatolenticular degeneration is accompanied by inflammation and acidification, but the mechanism underlying the development of fibrosis is currently less well studied (Wu et al., 2019). We have tried to prove that ASIC1a is activated by acidification caused by copper accumulation and plays a role in promoting fibrosis.

Acid-sensitive ion channels (ASICs) are cation channels activated by extracellular acidification. The ENaC/DEG family member has four coding gens: ASIC1, ASIC2, ASIC3, ASIC4, which encode seven subunit proteins: ASIC1a, ASIC1b, ASIC1b_2_, ASIC2a, ASIC2b, ASIC3, and ASIC4 have high permeability to Na^+^ and Ca^2+^. Among the seven subunits, ASIC1a is the only subunit that transports Ca^2+^. Studies have shown that various pathological conditions such as inflammation, ischemia, hypoxia, and tumors are accompanied by severe tissue acidification and low PH (Seki and Schwabe, 2015; Ortega-Ramirez et al., 2017). Therefore, there must be activation and opening of ASIC1a under these pathological conditions. ASIC1a activation causes the influx of extracellular Ca^2+^, which disrupts the calcium balance of the endoplasmic reticulum (Wu et al., 2014; Brown et al., 2016). The endoplasmic reticulum is the main site of calcium storage in the cell and participates in the regulation of Ca^2+^ signals in the cytoplasm. The endoplasmic reticulum plays an important role in signalling pathways. Disruption of calcium homeostasis can affect the synthesis and folding of endoplasmic reticulum proteins, as well as the posttranslational modification of proteins. It also causes endoplasmic reticulum stress (ERS) and activates the unfolded protein response (UPR) (Mori, 2009; Walter and Ron, 2011; Woehlbier and Hetz, 2011; Wu et al., 2016; Bergmann and Molinari, 2018). ERS can induce cell apoptosis by activating downstream apoptosis signaling molecules, and it can also regulate HSC activation and proliferation via the IRE1-XBP1 pathway (Yoshida et al., 2001; Huang et al., 2014). The most common mutant form of ATP7B still exhibits significant copper transport activity, but it is retained in the endoplasmic reticulum (ER) and is rapidly degraded during ERS (Forbes and Cox, 1998; Payne et al., 1998; de Bie et al., 2007; Parisi et al., 2018). It has recently been reported that the expression of this ATP7B mutant can activate the P38MAPK stress kinase pathway, which in turn triggers the quality control mechanism, resulting in inhibition in the ER and accelerated degradation (Chesi et al., 2016). Reducing ERS helps to retain ATP7B and thus reduces copper accumulation. It is crucial to explore the mechanism by which these factors function in the normalization of copper homeostasis in HLD.

Other studies have found that excessive copper levels can cause free radical-damage to nerve cells, activate MAPK signaling pathways, and activate protein and enzyme cascade signals in other cells. These signaling molecules can regulate nuclear transcription factors and gene expression, and ultimately cause intracellular calcium overload (Kadowaki et al., 2013; Darling and Cook, 2014; Kim et al., 2019; Li et al., 2019; Qiu et al., 2019). ASIC1a is activated, which promotes the activation of hepatic stellate cells and induces liver fibrosis. To further clarify whether ASIC1a is activated under copper loading, thus affecting the activation of hepatic stellate cells and whether the activation of ASIC1a may lead to the occurrence of ERS in HF-HSCs to accelerate the degradation of ATP7B. During the activation of ASIC1a, whether the P38MAPK pathway participates in and transmits stimulation signals. The experiments confirmed the expression of ASIC1a during HLD liver fibrosis, investigated the effect of regulating ASIC1a activity on HLD fibrosis and attempted to explain the role of the P38 MAPK pathway in HLD. This study provides the molecular experimental basis for the progress of HLD liver fibrosis injury mechanism. It provides specific therapeutic targets for the further development of drugs for the treatment of HLD.

## Materials and Methods

### Antibodies and Reagents

Fetal bovine serum (FBS) was purchased from Hangzhou Sijiqing Co., Ltd. DMEM high sugar medium was from HyClone Co. (United States). Quick Auto Neo Cu was provided by SHINO-TEST Co. (JPN). PcTX-1 was bought from Abcam Co. (United States). Lipofect3.0, TRIzol™ Reagent, Opti-MEM were from Invitrogen Co. (United States). Amiloride, Copper sulfate (CuSO_4_) were purchased from Sigma Co. (United States). D (-)-Penicillamine (PCA), P38MAPK inhibitor SB203580 were from TargetMol Co. (China). P38MAPK agonist Dehydrocorydaline was from MedChemExpress Co. (United States). Antibody against phospho-P38MAPK (Thr180/Tyr182) (1:1,000 times dilution) (catalog number: AF4001), P38MAPK(1:1,000 times dilution) (catalog number:AF6456), GRP78(1:1,000 times dilution) (catalog number:AF5366), XBP1(1:1,000 times dilution) (catalog number:AF5110), ATP7B(1:1,000 times dilution) (catalog number:AF0410), CCS(1:1,000 times dilution) (catalog number:DF3971), ASIC1a(1:1,000 times dilution) (catalog number:DF9198), and β-actin (1:5,000 times dilution) (catalog number:T0022) were from Affinity Co. (United States). Antibodies against α-SMA (1:5,000 times dilution) (catalog number: bs-0189R) and Collagen-Ι (1:1,000 times dilution) (catalog number: bs-0578R) were purchased from Bioss Co. (China). All siRNA sequences were designed and synthesized by Shanghai Han Hang Seng Material Technology Co., Ltd. RNA primers were designed synthesized by Shanghai Biology Engineering Corporation according to the serial number from Genbank, as shown in Table 1.

**TABLE 1:** Primer sequences of several genes for real-time PCR.

Gene	Forward primer	Reverse primer
β-actin	5′-GAG​CGC​AAG​TAC​TCT​GTG​TG-3՛	5՛-CCT​GCT​TGC​TGA​TCC​ACA​TC-3՛
ASIC1a	5՛-CGG​CTG​AAG​ACC​ATG​AAA​GG-3՛	5՛-AAG​GAT​GTC​TCG​TCG​GTC​TC-3՛
α-SMA	5՛-GAG​GGA​TCC​TGA​CCC​TGA​AG-3՛	5՛-CCA​CGC​GAA​GCT​CGT​TAT​AG-3՛
Collagen-Ι	5՛-ACC​TCA​GGG​TAT​TGC​TGG​AC-3՛	5՛-GAC​CAG​GGA​AGC​CTC​TTT​CT-3՛
GRP78	5′-CTG​TCA​GCA​GGA​CAT​CAA​GTT​C-3’	5′-TGT​TTG​CCC​ACC​TCC​ATT​ATC​A-3′
XBP1	5′-TCC​GCA​GCA​CTC​AGA​CTA​CG​-3′	5′-GGC​AAC​AGC​GTC​AGA​ATC​CA​-3′
CCS	5՛-ACA​GCT​GAC​CCC​TGA​GCG-3՛	5՛-ACA​GAG​CCA​AGG​TGA​GGT​C-3՛
ATP7B	5՛-GCC​AGC​ATT​GCA​GAA​GGA​AAG-3՛	5՛-TGA​TAA​GTG​ATG​ACG​GCC​TCT-3՛

### Establishment of Experimental Animals and Models

Sixty SPF grade male Wistar rats, weighing 160–200 g, were purchased from Jinan Pengyue Animal Breeding Co., Ltd. and raised in the school’s animal room (general-level breeding room) Pharmacy Anhui Medical University. The rats were housed adaptively for 1 week before the experiment. They were randomly divided into normal groups, copper load model groups, Amiloride groups, and PCA groups with 15 rats each. PCA was the positive control drug in the experiment. From the first day of the experiment, in addition to the normal group given ordinary feed, the other three groups were set up in the copper-loaded rat model, which our research group optimized according to the literature (Li et al., 1991). These groups were provided with 1.0 g/kg copper sulfate chow and 0.185% copper sulfate water for 12 weeks. The rats in each group ate equal amounts of food every day and drink freely. The feeding ecology of the normal group is the same as that of each model group. Starting from the 7th week, use intragastric gavage to intervene in each group. The normal group is given 0.02 ml/g saline once a day for six consecutive weeks, and the amiloride group is fed at 5 mg/kg Amiloride was intragastrically administered once a day for 6 weeks, and in the PCA group, PCA was administered once a day at 100 mg/kg for 6 weeks. During the process of modeling, observe the rat’s diet, water, activity, and mental status. After 12 weeks, the body weight was measured, and 10% chloral hydrate (0.4 ml/kg) was administered via the abdominal cavity for anaesthesia. Blood was collected from the abdominal aorta and centrifuged at 3,000 r/min to collect the serum. Part of the liver tissue was fixed with 4% neutral formaldehyde, and pathological and immunohistochemical experiments were performed. The remaining tissues were quickly placed in a freezer and stored at −80°C until testing. All the *in vivo* experiments were approved by the Animal Ethics Committee of Anhui Medical University and conducted by the Ethical Guidelines for the Care and Use System of Experimental Animals of Anhui Medical University.

### Histomorphological Examination of Rat Liver

Observe the survival status of each group of rats after the completion of the model. After fasting for 12 h, the rats were sacrificed, and the serum and liver were harvested for follow-up experiments. For pathological examination, the liver lobes of each group were collected and fixed with 4% neutral formaldehyde. The HE staining method was conducted. In brief, the tissue sections were deparaffinized with xylene, washed with various levels of ethanol, stained with haematoxylin for 7 min, and differentiated with 0.5–1% hydrochloric acid ethanol for 30 s after washing. Then, the sections were rewashed with eosin solution for 1 min, conventionally dehydrated, made transparent, and mounted. After scanning on the PANNORAMIC digital slice scanner of 3DHISTECH, the levels of inflammation and fibrosis in the liver tissues of each group were observed using Case Viewer software. Timm’s copper staining was performed after dewaxing in 0.5% ammonium sulfide for 5 min, and the sections were completely washed and incubated with 0.1 N hydrochloric acid for 2–3 min to remove iron and zinc sulfide solutions. After washing, the sections were placed in fresh solution A (5% silver nitrate aqueous solution) and five parts of filtered liquid B (2.0 g of bisphenol, 5.0 g of citric acid, 100 ml of distilled water). The conversion of copper sulfide into silver sulfide was conducted for 3 min, and then, the sections were washed with water, dehydrated, made transparent, mounted, and scanned. Then, copper deposition was observed.

### Serum Biochemical Index and Determination of Copper Content

An automatic biochemical analyser (Hitachi-3,100) was used to detect the content of copper ions in the rat serum samples according to the instructions of the copper ion biochemical kit. This instrument was also used to detect the content of ALT and AST in the rat serum samples according to the instructions of the kit.

### Immunohistochemistry

Using the streptavidin-biotin-peroxidase complex (SABC) method, the tissues are deparaffinized and repaired under high-pressure conditions. The blocking solution was added and incubated for 25 min. Then, the primary antibody (diluted at 1:100) was added dropwise, and the samples were washed with PBS. Next, the biotin-labelled secondary antibody (1:100) and streptavidin-biotin-peroxidase complex (DAB) were added dropwise for color development. The sections were washed with distilled water, dehydrated and mounted, and the stained cell sheets were scanned. For the positive specimens, PBS was used instead of the primary antibody as a negative control. The diffuse brownish-yellow coloration of HSC cytoplasm indicated positive target protein expression.

### Cell Culture and Growth Measurement

The rat hepatic stellate cell line HSC-T6 was obtained from Xiangya Medical College of Central South University. The rat hepatic stellate HSC-T6 cells were cultured in high-glucose DMEM containing 5% foetal bovine serum in an incubator at 37°C in 5% carbon dioxide. The HSCs were uniformly seeded in a 96-well plate at a density of 5 × 10^3^ cells per milliliter, and after the cells assumed their normal morphology (usually 24 h), 100 μL medium per well was replaced with medium containing 1% serum as the negative (0 µM copper sulfate) control. To other wells, medium containing 20, 40, 80, and 100 µM copper sulfate solution was added, and the cells were incubated for 6, 12, and 24 h. Then, 10 µL of CCK-8 solution was added to each well, the cells were incubated for 2–4 h in an incubator, and the absorbance at 490 nm was measured with a microplate reader. The absorbance value indicated the viability and survival of the cells. The IC_50_ of HSC-T6 cells treated with copper was calculated based on the absorbance value and was used as the concentration of copper for stimulating the cell model in subsequent experiments.

### Immunofluorescence

The treated cells were aspirated to remove the medium and washed with PBS three times. The cells were fixed in 4% paraformaldehyde for 10 min and then treated with PBS containing 10% bovine serum albumin (BSA) closed for 1 h. The cells were incubated with a primary antibody against phospho-P38MAPK (1:100 dilution) at 4°C overnight and then incubated with FITC-conjugated anti-rabbit IgG in the dark at 37°C for 1 h. After washing with PBS, the nuclei were stained with 4,6-dimidamino-2-phenylindole (DAPI) for 5 min in the dark and then examined by an inverted fluorescence microscope (Olympus, Tokyo, Japan).

### Small Interference RNA Transfection

Hepatic stellate cells (2 × 10^5^) were seeded into 6-well plates and cultured in a humidified incubator at 37°C and 5% CO_2_ for 24 h. According to the manufacturer’s protocol, cells were transfected with a negative control siRNA (NC-siRNA) and siRNA targeting ASIC1a by Lipofect3.0 and Opti-MEM. The sequence of targeted ASIC1a-siRNA was as follows: sense:5՛–GCGUGAAUUCUACGACAGA–3՛; Antisense: 5՛–UCU​GUC​GUA​GAA​UUC​ACG​C –3՛. The sequences of negative control siRNA were: sense: 5՛–UUCUCCGAACGUGUCACGUdTdT–3՛; Antisense: 5՛–ACGUGACACGUUCGGAGAAdTdT–3՛. The transfection efficiency was tested using Western Blot and qRT-PCR technology. After the transfection, discard the transfection reagent and change to a medium containing 20 μm copper sulfate. After culturing for 24 h, collect the cells for subsequent experiments.

### Western Blot Analysis

The treated HSCs were incubated with 500 μL RIPA cell lysis buffer and 5 μL PMSF on ice for 30 min to lyse the cells. Then, the cells were centrifuged at 12,000 × g at 4°C for 30 min, the supernatant was removed, and the BCA protein detection kit was used for the quantitative analysis of the protein concentrations. After polyacrylamide gel electrophoresis, the proteins were transferred to a polyvinylidene fluoride (PVDF) membrane by electroporation. The membranes were incubated with 5% skimmed milk for 3 h and then incubated overnight at 4°C with a primary antibody specific for β-actin. After washing three times in Tris-buffered saline (TBS)/Tween-20, the membrane was incubated with horseradish peroxidase-conjugated rabbit anti-mouse (1:10,000, OriGene Technologies, Beijing, China) or goat anti-rabbit immunoglobulin G (IgG; 1:10,000, OriGene Technologies, Beijing, China) antibodies at 37°C for 1 h. The membranes were washed three times with TBS/Tween-20, and then an enhanced chemiluminescence (ECL) kit (Thermo Scientific, United States) was used for detection. β-actin served as the internal reference, and the image analysis software ImageJ was used to analyse the protein bands. The ratio of the target protein expression to β-actin expression is presented as the relative expression level of the target protein.

### Real-Time Quantitative Polymerase Chain Reaction

The total cellular RNA was extracted using TRIzol™ reagent. Resuspend the extracted RNA pellets in 10 µl of ribonuclease-free water to dissolve the RNA. RNA content and purity were determined spectrophotometrically. According to the manufacturer’s instructions, cDNA was generated from purified RNA using the PrimeSciptTM RT reagent Kit (Takara). The cDNA analysis using SYBR Premix Ex Taq II (TAKARA) performed on Bio-Rad CFX connect fluorescent quantitative PCR system with 96 wells. All PCR runs were performed in triplicate, and the results were averaged. The gene expression levels and PCR efficiency, along with its standard error, were analysed, and the efficiencies were nearly 100%, allowing the use of the 2^−△△Ct^ method to calculate the relative gene expression levels. β-actin was used as the reference gene for normalization.

### Enzyme-Linked Immunosorbent Assay of IL-6 and TNF-α

The samples were pretreated, and blood samples were collected from rats by using a tube without pyrogen and endotoxin. The tubes were incubated at room temperature for 2 h to separate the serum. The samples were centrifuged at 3,000 × g for 15 min at 4°C, the supernatant (serum) was carefully collected, it was measured immediately or stored in a freezer at −20°C. The cell culture supernatants were transferred to centrifuge tubes and centrifuged at 1,000 × g for 20 min to remove cell debris and impurities. The supernatants were collected for later use. The samples were stored at −20 or −80°C. Perform enzyme-linked immunosorbent assay according to the instructions of the rat IL-6 and TNF-α kits. The optical density (OD value) of each well was measured at the 450 nm wavelength with a microplate reader, and the standard curve was drawn with the OD values of the standard samples. This standard curve was used to determine the concentration of the samples.

### Statistical Analysis

The data were processed by GraphPad Prism 7 analysis software. Comparisons between the sample means of multiple groups was performed by one-way analysis of variance (one-way ANOVA), and comparisons of independent sample means was performed by t-test analysis. All the data were obtained from three independent experiments that were performed in triplicate and are presented as the mean ± standard error. *p* < 0.05 indicates that the difference is significant.

## Results

### Copper Loading Induces the Activation of HSC-T6 and Activates ASIC1a

**FIGURE 1:**
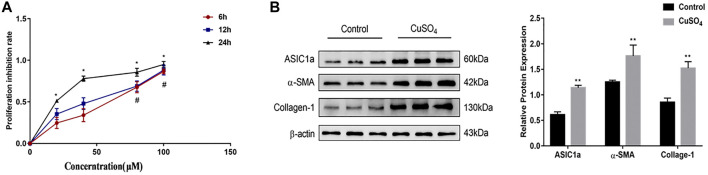
**(A)** Rates of HSC-T6 cell growth inhibition after treatment with different concentrations of copper sulfate for different times. The concentration of copper sulfate was set to 0, 20, 40, 80, and 100 µM. The data are shown as representative mitochondrial dehydrogenase activity as measured by CCK-8 assay. Data are expressed as the mean ± SEM (*n* = 3). ^*^ *p* < 0.05 vs. Control group (24 h); ^#^ *p* < 0.05 vs. Control group (6 & 12 h) **(B)** Activation of HSC-T6 cells and expression of ASIC1a in copper-treated cell models. The experimental group was repeated three times, and Western blotting analysis and densitometric quantification of ASIC1a, α-SMA, and Collagen-I protein levels in HSCs were performed. Statistical analyses were performed using t-test. Data are expressed as the mean ± SEM (*n* = 6). ^*^ *p* < 0.05, **p < 0.01 vs. Control group.

Our previous study showed that ASIC1a expression was increased in acid-induced hepatic stellate cells. Acidification of the HSC-T6 culture environment to PH 6.0 opened the ASIC1a channel and increased its mRNA and protein expression. In this study, the CCK-8 method was used to detect the viability of the HSCs cultured with different concentrations of copper sulfate solution after 6, 12, and 24 h (Figure 1A). GraphPad Prism 7 was used to graphically determine the concentration of CuSO_4_ required to reduce cell proliferation by 50% (IC_50_). The results showed that 20 µM copper sulfate was closest to the IC50 at 24 h, and thus, a cell model of copper sulfate treatment was established. The treated cells were collected, and Western blotting was used to detect the expression of ASIC1a and the fibrosis-related proteins α-SMA and Collagen-Ι. The results showed that the level of fibrosis was significantly increased after copper treatment (*p* < 0.01), suggesting that copper accumulation can activate acid-sensitive ion channel 1a and activate hepatic stellate cells (Figure 1B).

### Inhibition of ASIC1a Improves Cu-Induced HSCs Activation and ERS

**FIGURE 2:**
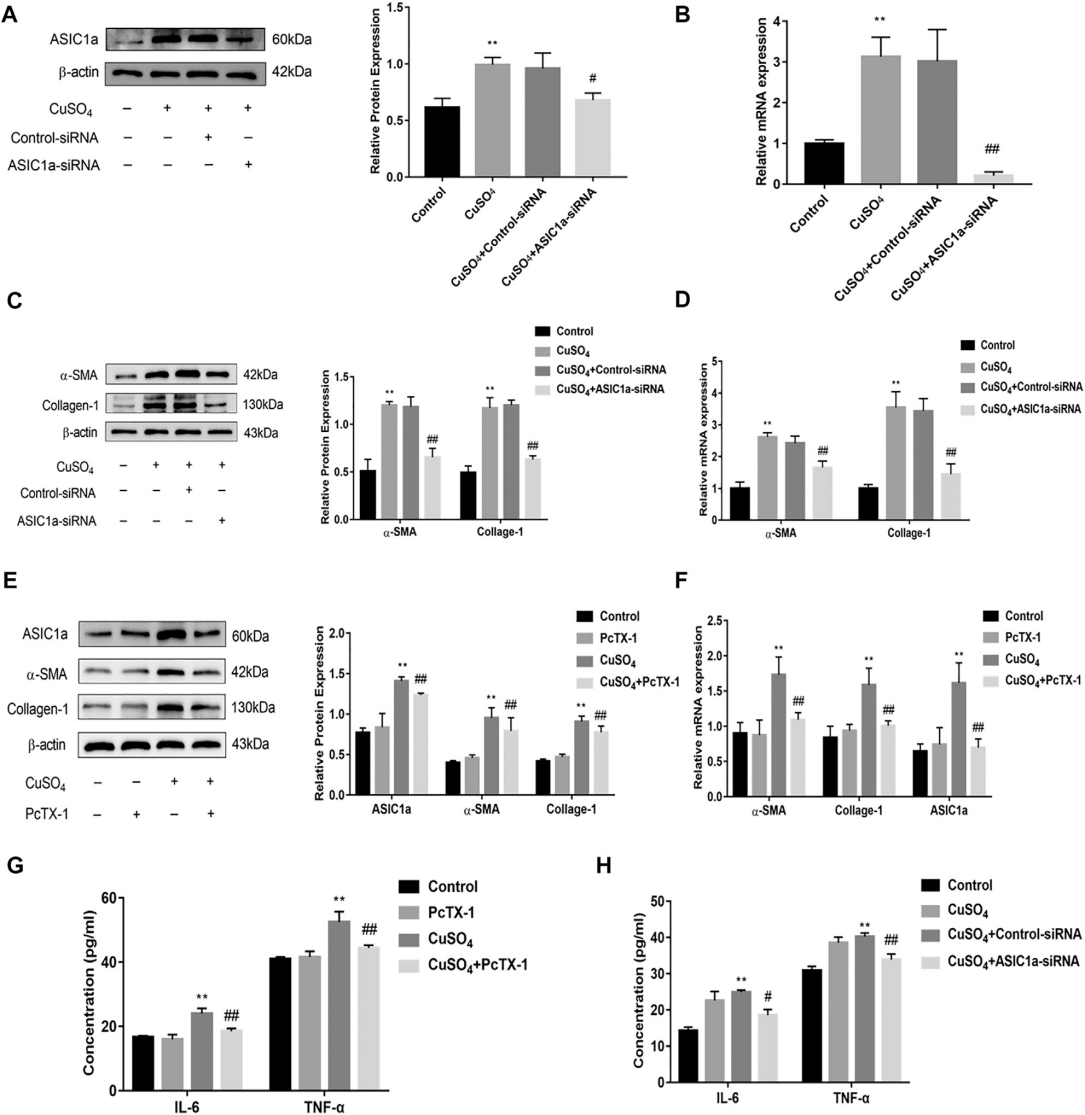
Effect of ASIC1a-siRNA transfection and PcTX-1 on ASIC1a, α-SMA, and collagen-Ι expression in HSC-T6 cells **(A)** Western blotting analysis and densitometric quantification of ASIC1a protein levels in HSCs after transfection of ASIC1a-siRNA; **(B)** mRNA levels of ASIC1a in HSCs after transfection of ASIC1a-siRNA **(C)** Expression of the fibrotic proteins in HSCs after transfection of ASIC1a-siRNA; **(D)** mRNA levels of α-SMA and Collagen-I in HSCs after transfection of ASIC1a-siRNA **(E)** Western blotting analysis and densitometric quantification of α-SMA, and Collagen-I protein levels in HSCs under PcTX-1; **(F)** mRNA levels of α-SMA, and Collagen-I in HSCs treated with PcTX-1 **(G)** Levels of IL-6 and TNF-α in cell medium after treatment with PcTX-1; **(H)** Levels of IL-6 and TNF-α in the cell medium after transfection with ASIC1A-siRNA. Statistical analyses were performed using t-test. Data are expressed as the mean ± SEM (*n* = 4). ^*^ *p* < 0.05, **p < 0.01 vs. Control group; ^#^ *p* < 0.05, ^##^ *p* < 0.01 vs. CuSO_4_ group.

**FIGURE 3:**
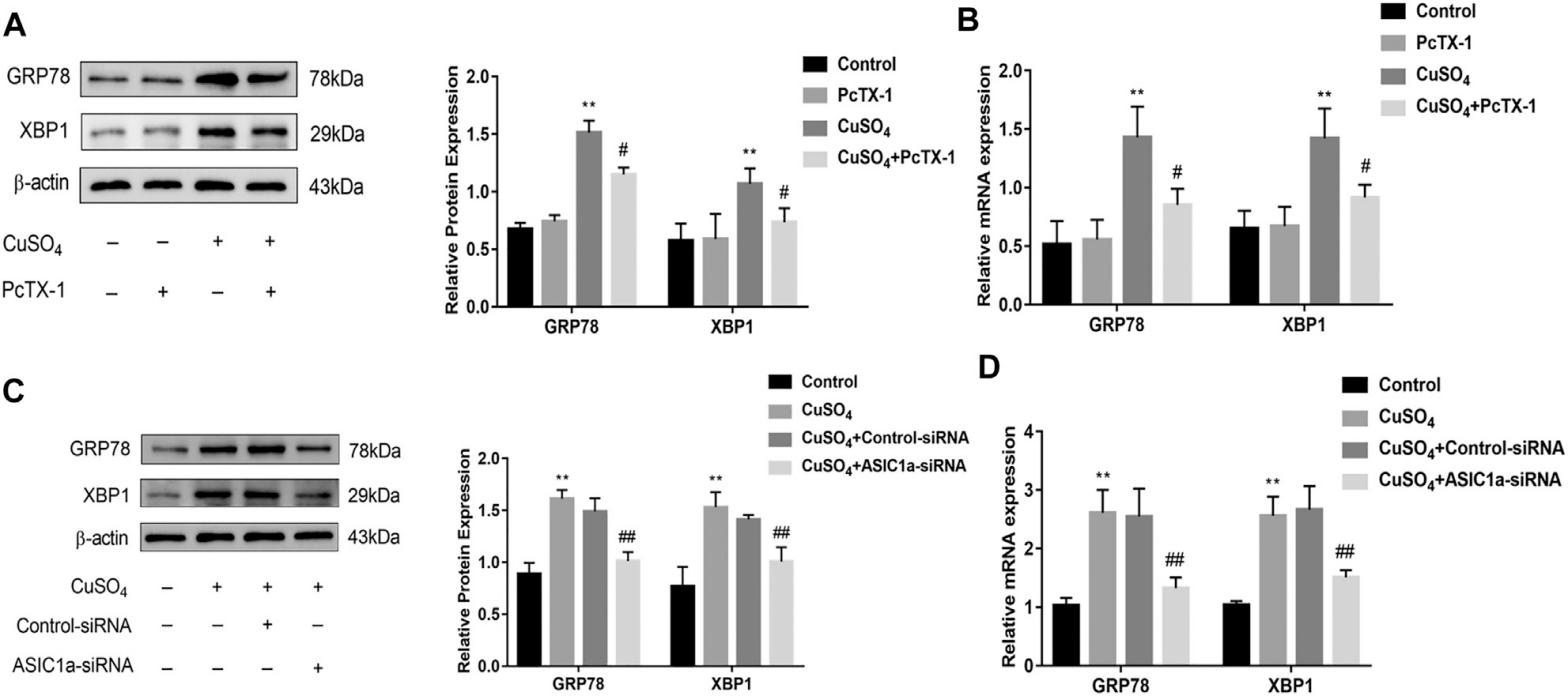
The effect of regulating the expression of ASIC1a on ERS in copper-treated HSC-T6 cells **(A**) Western blotting analysis and densitometric quantification of GRP78, and XBP1 protein levels in HSCs treated with PcTX-1; **(B)** mRNA levels of GRP78, and XBP1 in HSCs treated with PcTX-1 **(C)** Western blotting analysis and densitometric quantification of GRP78, XBP1 protein levels in HSCs transfected with ASIC1a-siRNA; **(D)** mRNA levels of GRP78, XBP1 in HSCs transfected with ASIC1a-siRNA. Statistical analyses were performed using t-test. Data are expressed as the mean ± SEM (*n* = 4). ^*^ *p* < 0.05, **p < 0.01 vs. Control group; ^#^ *p* < 0.05, ^##^ *p* < 0.01 vs. CuSO_4_ group.

**FIGURE 4:**
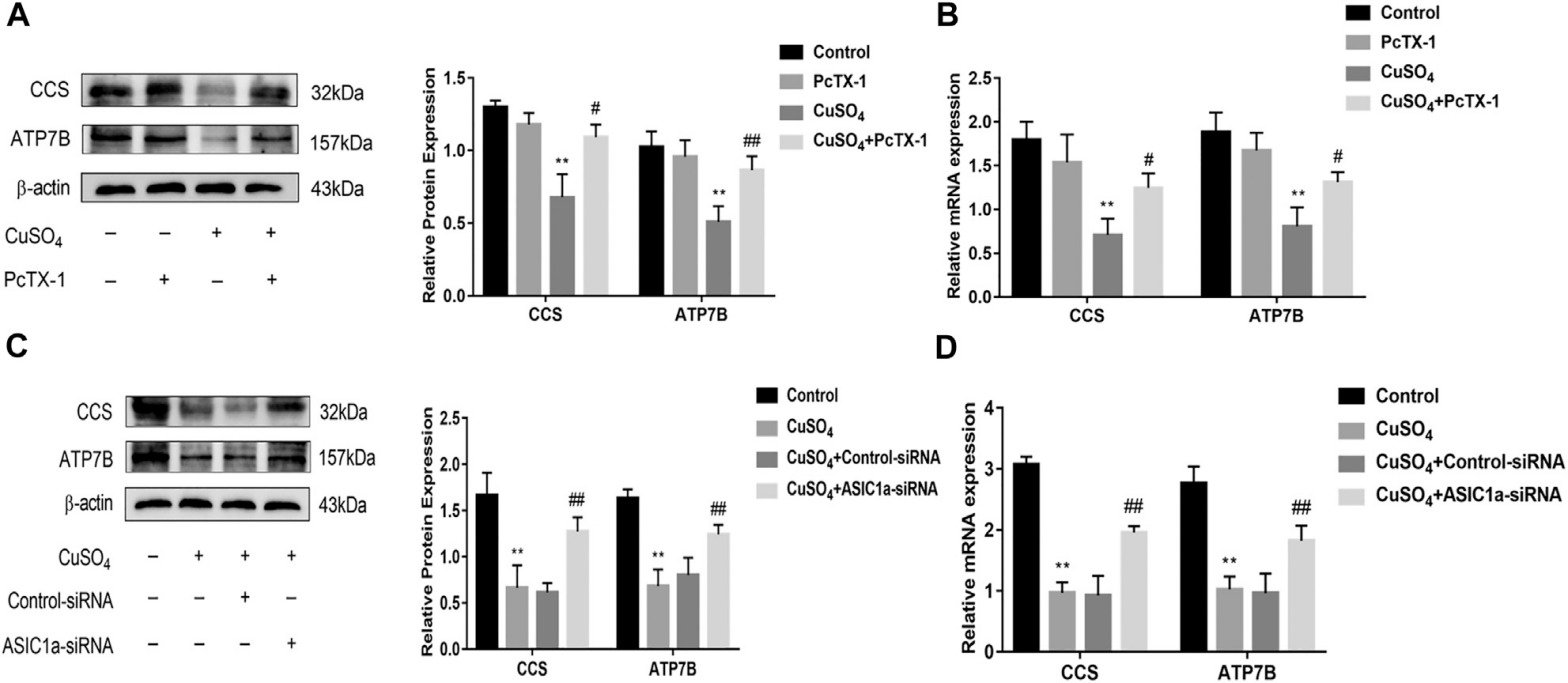
Effects of inhibition and silencing of ASIC1a on copper metabolism-related proteins **(A)** Western blotting analysis and densitometric quantification of ATP7B, and CCS protein levels in HSCs treated with PcTX-1; **(B)** mRNA levels of ATP7B, and CCS in HSCs treated with PcTX-1 **(C)** Effect of ASIC1a-siRNA transfection on the expression of copper metabolism-related proteins ATP7B and CCS; **(D)** mRNA levels of ATP7B and CCS in HSCs after transfection with ASIC1a-siRNA. Statistical analyses were performed using t-test. Data are expressed as the mean ± SEM (*n* = 4). ^*^ *p* < 0.05, **p < 0.01 vs. Control group; ^#^ *p* < 0.05, ^##^ *p* < 0.01 vs. CuSO_4_ group.

Excessive copper accumulation can cause ERS and ATP7B degradation in hepatic stellate cells. These processes are accompanied by the expression of inflammatory cytokines. Studies have shown that ERS is involved in the activation of HSCs and that ASIC1a plays a role in this process. ASIC1a-siRNA and Control-siRNA were mixed with Lipofect3.0 at a ratio of 1:1 and then transfected into rat hepatic stellate cells. After ASIC1a-siRNA was transfected into the rat hepatic stellate cells, the mRNA and protein expression levels of ASIC1a in the cells were decreased, and the differences were statistically significant (*p* < 0.05) (Figures 2A,B). In addition, the protein expression of ASIC1a in the hepatic stellate cells of the Control-siRNA group was not significantly different from that of the copper load model group. HSCs were preincubated with the ASIC1a-specific inhibitor psalmotoxin-1 (PcTx-1; 100 nM) for 1 h, and then, the medium was replaced with medium containing copper sulfate for 24 h. The Western blotting and qRT-PCR results showed that after blocking ASIC1a with the specific inhibitor PcTX-1 or transfecting cells with specific ASIC1a-siRNA to knock down ASIC1a expression, the expression of ASIC1a and the activation marker proteins α-SMA and Collagen-Ι was significantly reduced. Compared with the copper-loaded group, significant differences suggest that ASIC1a promoted the copper-induced HSC activation (Figures 2C–F). Some studies have shown that inflammatory cytokine-mediated inflammatory responses may also be involved in tissue damage in patients with HLD. The ELISA results showed that the levels of IL-6 and TNF-α in the medium were significantly increased in the CuSO_4_-induced group compared with the normal group, and the concentration decreased after the expression of ASIC1a was inhibited (Figures 2G,H). After downregulating ASIC1a expression, the expression of the ERS marker proteins GRP78 and XBP1 was significantly downregulated, and the difference was statistically significant (*p* < 0.05) (Figures 3A–D), indicating that ASIC1a may regulate the occurrence of ERS in copper-treated HSCs. Endoplasmic reticulum-associated degradation (ERAD) degrades the ATP7B protein, and copper accumulation increases this process. The expression of superoxide dismutase copper companion (CCS) may vary depending on the amount of copper in the environment. CCS contains 270–300 amino acid residues and specifically transfers copper ions to SOD1 in the form of homologous dimers, further promoting SOD1 disproportionation and protecting cells from oxidative free radical-mediated damage; thus, CCS is very important for maintaining intracellular copper homeostasis. After transfection with ASIC1a-siRNA, or intervention with inhibitor PcTX-1, we used Western Blot analysis to detect the expression of copper metabolism-related proteins CCS and ATP7B (Figures 4A–D). The results showed that after ASIC1a was downregulated, the decrease in the expression of ATP7B and CCS caused by copper load was significantly ameliorated. The difference was statistically significant (*p* < 0.05). The qRT-PCR test results of related indicators were consistent with the Western Blot results, indicating that ASIC1a may regulate the quality control of the ER to reduce the degradation of ATP7B and reduce the accumulation of copper in cells.

### Copper-Induced ASIC1a Regulates Copper Transport Through the P38MAPK Signaling Pathway

**FIGURE 5:**
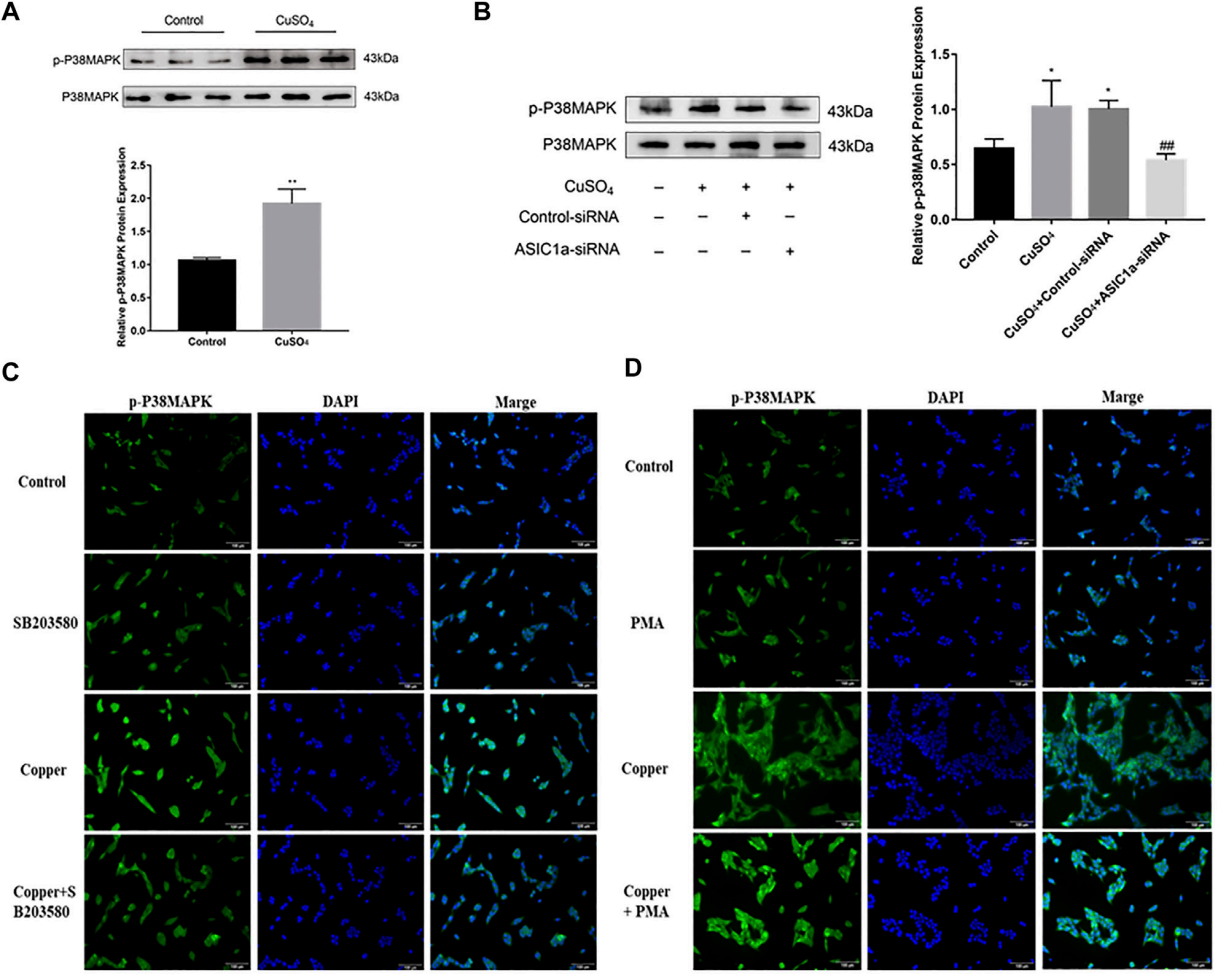
ASIC1a is involved in regulating the phosphorylation of P38MAPK under copper accumulation conditions **(A)** Protein expression of phosphorylated P38 MAPK in copper-treated HSC-T6 cells. Data are expressed as the mean ± SEM (*n* = 6). *p < 0.05, **p < 0.01 vs. Control group **(B)** Copper-induced changes in the level of phosphorylated P38MAPK in HSCs transfected with ASIC1a-siRNA. Data are expressed as the mean ± SEM (*n* = 4). ^*^ *p* < 0.05, **p < 0.01 vs. Control group; ^#^ *p* < 0.05, ^##^ *p* < 0.01 vs. CuSO_4_ group **(C)** Immunofluorescence detection of copper-induced phosphorylation of P38MAPK in hepatic stellate cells treated with the P38 MAPK pathway inhibitor SB203580. (magnification 200×) **(D)** Immunofluorescence detection of copper-induced P38 MAPK phosphorylation in hepatic stellate cells treated with the P38 MAPK pathway agonist dehydrocorydaline (PMA) (magnification 200×).

**FIGURE 6:**
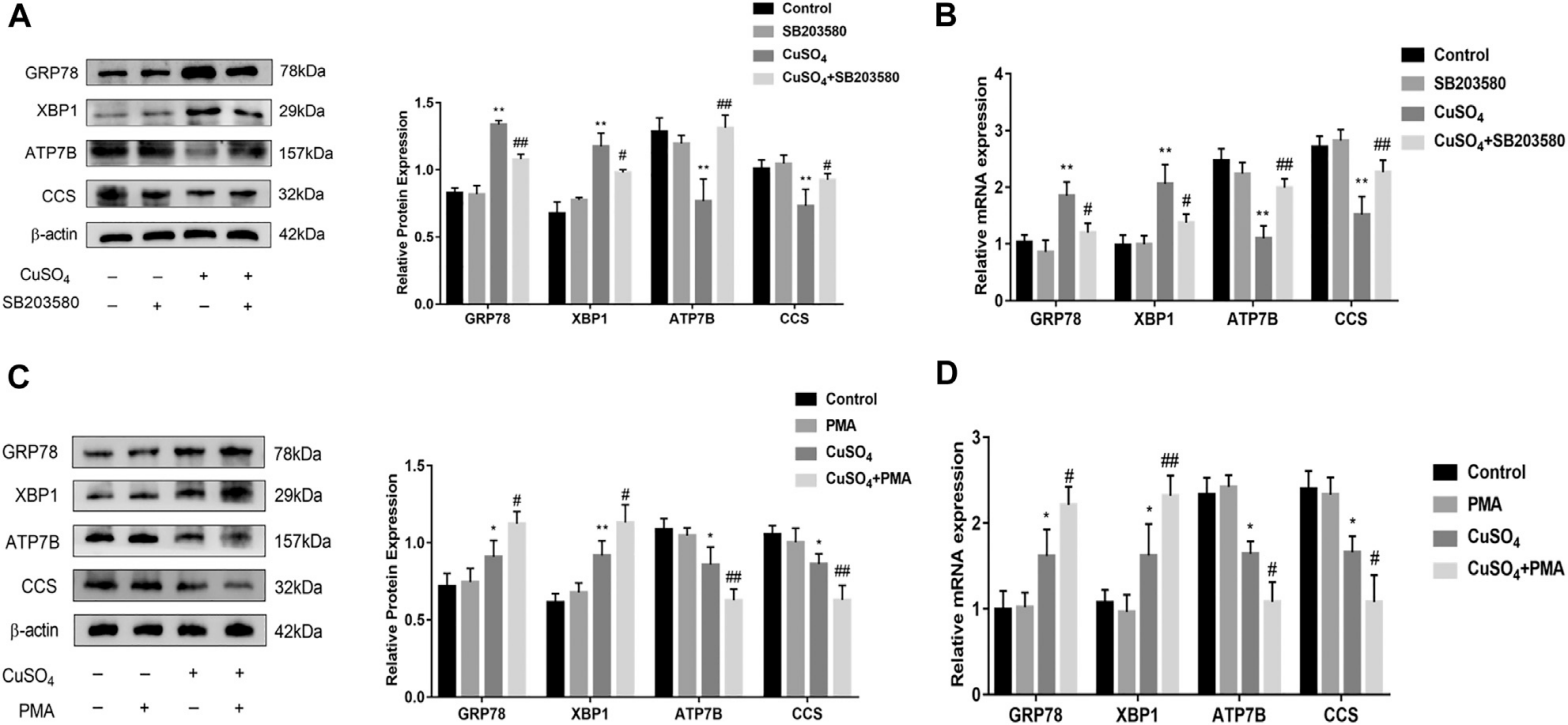
Inhibition and activation of the P38MAPK pathway regulates endoplasmic reticulum stress and copper transport (**A**) Western Blot detects the protein expression of GRP78, XBP1, copper transporter ATP7B, CCS after treatment with the P38MAPK pathway inhibitor SB203580; (**B**) The mRNA levels of GRP78, XBP1, ATP7B, CCS in cells treated with the SB203580 (**C**) Western blotting analysis and densitometric quantification of GRP78, XBP1, ATP7B, and CCS protein levels in HSCs treated with the P38MAPK pathway agonists dehydrocorydaline; (**D**) mRNA levels of GRP78, XBP1, ATP7B, and CCS in HSCs treated with dehydrocorydaline. The data are expressed as the mean ± SEM (*n* = 4). ^*^ *p* < 0.05, **p < 0.01 vs. Control group; ^#^ *p* < 0.05, ^##^ *p* < 0.01 vs. CuSO_4_ group.

It has been reported in the literature that P38MAPK can activate the ERS under copper accumulation conditions. We hypothesized that if the ERS mediated by ASIC1a under conditions of high copper requires the participation of the MAPK signalling pathway, downregulating the protein level of ASIC1a in HSCs would decrease the activation of signalling pathways. Western blotting analysis was used to detect the protein expression of phosphorylated P38MAPK in HSC-T6 cells after copper treatment. The results showed that *p*-P38 MAPK expression was significantly increased after copper treatment, suggesting that the P38 MAPK pathway may be involved in copper sulfate-induced HSC activation (Figures 5A,B). After the expression of ASIC1a was downregulated by transfection with ASIC1a-siRNA, the level of phosphorylated P38MAPK was reduced, suggesting that ASIC1a regulates the occurrence of ERS through the P38MAPK pathway. To further explore the role of the P38MAPK signaling pathway in ERS, P38MAPK signaling pathway inhibitors and agonists were used to regulate the level of phosphorylation P38MAPK, and then, whether ERS was improved was determined. Firstly, immunofluorescence was used to observe the effects of inhibitors and agonists on phosphorylation of the P38MAPK signaling pathway in HSCs (Figures 5C,D). After pretreatment of cells with inhibitor SB203580 (10 µM) for 24 h to block the P38MAPK pathway (Leelahavanichkul et al., 2014), the Western Blotting and qRT-PCR results showed that compared with the copper load group, the expression of ERS marker proteins GRP78 and XBP1 were significantly decreased, and the expression of ATP7B and superoxide dismutase CCS was relatively increased. The results were statistically significant (*p* < 0.05) (Figures 6A,B). Pretreatment with dehydrofumarine (100 nM) for 24 h can exacerbate ERS by activating the P38MAPK signalling pathway (Mugami et al., 2017). Compared with those in the copper load group, the GRP78 and XBP1 protein levels in the dehydrocorydaline group were significantly increased, while the expression of ATP7B and CCS was decreased (Figures 6C,D). These results indicated that the P38MAPK pathway accelerated the degradation of ATP7B by activating endoplasmic reticulum quality control and the unfolded protein response, leading to the exacerbation of copper accumulation, which was regulated by ASIC1a activation.

### Calcium Channel Blockers Amiloride (ASIC1a Nonspecific Inhibitor) Improved Copper Accumulation and Fibrosis in the Liver of Copper-Loaded Rats

**FIGURE 7:**
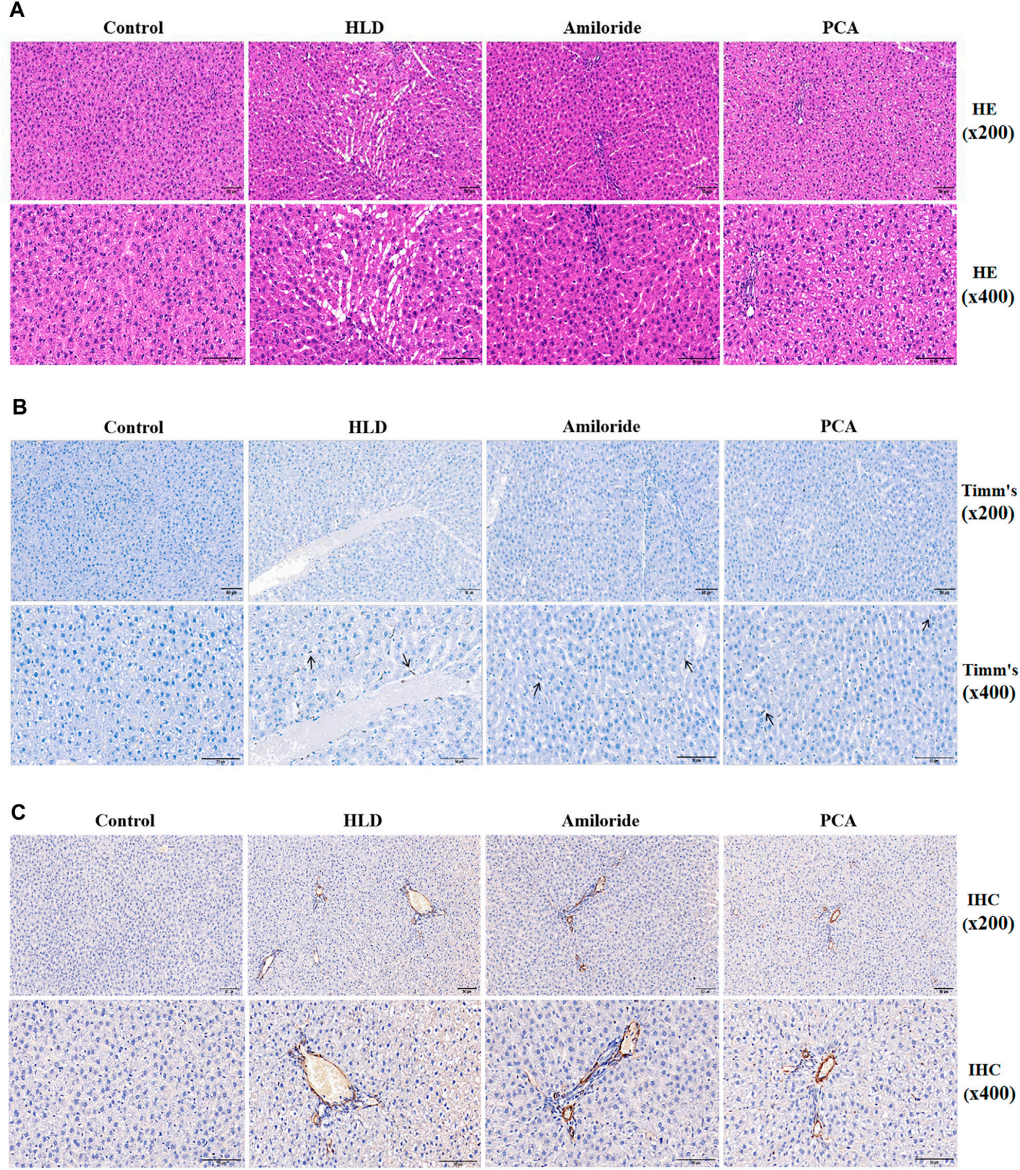
Observation of pathological changes and α-SMA expression induced by Amiloride and D-penicillamine in the livers of copper-treated rats **(A)** Histopathological results of liver tissues of rats in each group (scale bar, 50 µM) **(B)** Timm’s copper staining was used to observe the deposition of copper particles in the liver tissues of rats in each group (scale bar, 50 µM) **(C)** The immunohistochemical method was used to detect α-SMA protein expression in liver tissues of rats in each group (scale bar, 50 µM).

After establishment of the model, the serum copper contents of the rats in each group were detected by an automatic biochemical analyser (Table 2). The copper content in the model group was more than double that in the normal group (*p* < 0.01), suggesting the establishment of a copper-loaded rat model. Compared with the model group, the serum copper levels of the Amiloride group and PCA group were significantly reduced (*p* < 0.01), and there was no significant difference between these two groups. At the same time, the activity of rat serum ALT and AST were tested. The results showed that compared with the normal group, the serum ALT and AST activities of the model group, Amiloride group, and PCA group increased (*p* < 0.05). Compared with the model group, serum AST decreased in the Amiloride and PCA groups (*p* < 0.05). This result shows that as copper is deposited in liver cells, serum ALT and AST activities also increase. The results of HE staining showed that the structure of liver lobules in the normal group was intact and clear, and the liver cells arranged orderly manner (Pronicki, 2017). In the model group, fiber cords formed multiple pseudo-lobules, aggravation of fatty degeneration and necrosis, and cytoplasm observed vacuolation. Fibrosis in the Amiloride group was improved, necrosis and steatosis were reduced. In the PCA group, hepatocytes had extensive granular degeneration, improved fibrosis, and cytoplasm could see tiny red granules. Compared with the model group, each treatment group exhibited less degeneration and necrosis of liver cells, reduced pathological changes, and more complete cell structures (Figure 7A). Timm’s positive copper staining shows the uneven distribution of black particles or clump-like material deposits. The most marked accumulations of copper were found within the hepatocytes of the centrilobular zones, deposited in the perinuclear region or at the biliary pole of hepatocytes, or extended to the periportal zones. A single cell undergoing necrosis is occasionally seen enveloped in copper granules. Positive copper staining was observed in the model group and each treatment group, and the deposition of copper particles in the treatment group was decreased, with no difference between the groups (Figure 7B). According to the immunohistochemical results, the positive signal of the α-SMA protein was brown, and positive cell expression was observed in the model group and each treatment group. The number of positive cells in the model group is significantly higher than that in the blank group. The portal area of fibrogenesis, the peripheral blood vessels, and the cells around the hepatic sinuses near the fibrous cords also have positive expression. The number of positive cells in the D-penicillamine group and Amiloride group was significantly reduced compared with that in the model group, but there was no significant difference between the D-penicillamine and Amiloride groups (Figure 7C).

**TABLE 2:** Activities of ALT and ALT, and concentrations of copper in sera of rats.

Group	Activities		Concentrations of copper(µg/dl)
	ALT(U/L)	AST(U/L)	
Normal	22.80 ± 9.91	55.62 ± 19.59	46.76 ± 4.63
HLD	69.50 ± 62.47^a^	105.50 ± 51.26^a^	106.10 ± 15.69^a^
Amiloride	28.14 ± 13.43^b^	54.71 ± 15.79^b^	63.17 ± 5.33^b^
PCA	27.00 ± 14.32^b^	49.86 ± 14.30^b^	63.17 ± 5.33^b^

a Represents a statistically significant difference from the control group (*p* < 0.01, respectively).

b Represents a statistically significant difference between the drug administration group and the modeling group, respectively, (*p* < 0.01).

### Effect of Calcium Channel Blocker Amiloride on Fibrosis and Copper Transport in Copper-Loaded Rats

**FIGURE 8:**
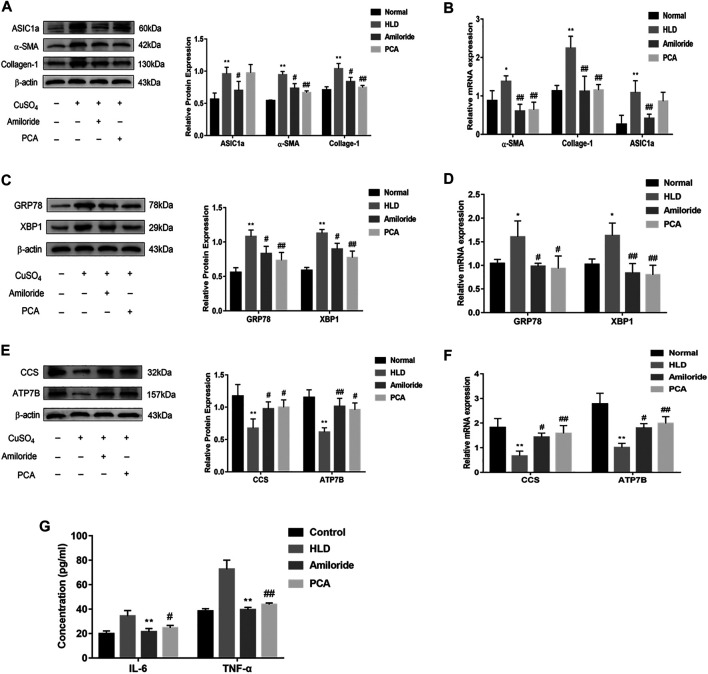
Protein and mRNA expression in liver tissues of Wistar rats treated with copper. Western blotting analysis and densitometric quantification of ASIC1a, α-SMA, and Collagen-I **(A)**, ERS marker protein GRP78, XBP1 **(C)**, ATP7B, and CCS **(E)** in the Wistar rats in each group. Changes in the gene expression of ASIC1a, α-SMA, and Collagen-I **(B)**, the ERS marker protein GRP78, XBP1 **(D)**, ATP7B, and CCS **(F)** in the liver tissues of copper-treated rats after amiloride and penicillamine treatment **(G)** Serum levels of IL-6 and TNF-a in Wistar rats. Statistical analyses were performed using a *t*-test. The data are expressed as the mean ± SEM (*n* = 4). ^*^ *p* < 0.05, **p < 0.01 vs. Normal group; ^#^ *p* < 0.05, ^##^ *p* < 0.01 vs. HLD group.

Western blotting showed that the protein level of ASIC1a was significantly different between the groups. ASIC1a was highly expressed in the liver tissue of copper-loaded rats. The expression of ERS marker proteins GRP78 and XBP1 were also significantly increased in the copper-loaded group compared with the normal group. The model group exhibited higher expression than the normal group. The relative expression was significantly increased. The relative expression of proteins in the D-penicillamine group and the Amiloride group was significantly decreased compared with that in the model group (Figures 8A,C) (*p* < 0.01). There was no significant difference between the Amiloride group and the D-penicillamine group. The expression of ATP7B and superoxide dismutase CCS was significantly decreased after copper treatment, and the expression in the D-penicillamine group and amiloride group was increased relative to that in the model group (*p* < 0.01) (Figure 8E). It is suggested that Amiloride inhibits the activation of ASIC1a by blocking calcium channels, reduces the occurrence of ERS, increases the expression of ATP7B and CCS, and ameliorates the accumulation of copper. The changes in gene levels of copper-loaded rats using qRT-PCR detect. The expression of ASIC1a, α-SMA, and Collagen-I in the copper-loaded group were significantly increased compared with that in the normal group (Figure 8B). The ERS-related factors GRP78 and XBP1 also showed high expression (Figure 8D), and the expression of ASIC1a was inhibited in the Amiloride group but not in the PCA group, while the expression of α-SMA, Collagen-1, GRP78, and XBP1 in the liver tissues of these two groups was reduced compared with that in the model group (*p* < 0.05). ATP7B and CCS are highly expressed in liver tissues of normal rats but significantly decreased in the model group, and the expression of the D-penicillamine group and Amiloride group was relatively increased compared with the model group (*p* < 0.01) (Figure 8F). The ELISA results showed that compared with the normal group, the copper-loaded group exhibited significantly increased serum IL-6 and TNF-α levels, suggesting that the inflammatory response is involved in the process of hepatolenticular degeneration (Figure 8G).

## Discussion

Hepatolenticular degeneration, also known as Wilson’s disease (WD), is an autosomal recessive inherited disorder of copper metabolism, and it has a worldwide incidence of 1/30,000–1/50,000 (Xie and Wu, 2017; Członkowska et al., 2018; Sandahl et al., 2020). The abnormal gene is located on chromosome 13 q14 ∼ q21; this gene is also known as the ATP7B gene, and it encodes a P-type copper transporter ATPase (ATP7B protein) (Chang and Hahn, 2017). Copper is transported to ceruloplasmin in the reverse network of the Golgi apparatus and transported to vesicles for export into the bile when copper levels become excessive. Mutations in the ATP7B gene cause the ATP7B enzyme to lose its function, and the dysregulation of copper transport leads to the accumulation of copper in the liver and brain of patients. Accumulation of copper in the cytoplasm of hepatic epithelial parenchymal cells leads to necrosis of the liver. At the same time, large amounts of copper are released into the blood and deposited in tissues such as the brain, kidney, and cornea. The clinical features are progressive worsening of liver cirrhosis, extrapyramidal symptoms, psychiatric symptoms, kidney damage, and corneal pigment ring (Kayser-Fleischer ring, K-F ring). Serum copper in HLD patients binds to proteins in tissues and deposits in the liver, developing liver fibrosis and then developing liver cirrhosis (Hedera, 2017; Lorincz, 2018). If liver fibrosis can be detected early and treated in time, it can still be reversed. Therefore, intervention and treatment for HLD patients with liver fibrosis are of great significance (Huster and Lutsenko, 2007; Zischka and Lichtmannegger, 2014). In this experiment, a copper-loaded Wistar rat model was created by adding copper sulfate to the diet. We can use Timm’s staining to observe many copper particles deposits in the liver after 12 weeks of copper load, which appear as intracytoplasmic small black or greenish-black granules in the cytoplasm. It can be detected in liver cells around the portal vein. The automatic biochemical analyzer detected that the serum copper content of rats in the copper-loaded group was more than twice that of the normal group. Rats serum ALT and AST activities also increased after copper load, indicating that the excessive deposition of copper ions in the liver caused the damage of hepatocytes. There are also literature studies in patients with hepatolenticular degeneration and fibrosis patients with hepatic lobule disorders; the fibrotic lobule disorder may lead to the interruption of the bile ducts and then the bile flow disorder. In such patients, copper particles were also found to be deposited in the hepatocytes and perinuclear cytoplasm. It seems that copper staining also provides valuable information for assessing the progression of chronic liver diseases such as fibrosis (Miyamura et al., 1988). HE staining revealed characteristics of fibrosis. In addition, the total protein and RNA extracted from the rat liver tissues were examined, and the results showed that the expression of ATP7B and superoxide dismutase copper molecular chaperone CCS was significantly reduced after copper accumulation, suggesting a copper transport disorder. More importantly, this study found, for the first time the expression of ASIC1a in the liver tissues of copper-treated rats and that copper deposition and hepatolenticular fibrosis can be ameliorated by inhibiting ASIC1a.

Acid-sensitive ion channels are a type of cation channel activated by extracellular acid. The channels are permeable to Na^+^ and Ca^2+^ when they are open (Lingueglia et al., 1997; Holzer, 2009; Qadri et al., 2012). Under normal conditions, ASIC1a is mainly located on the nuclear and endoplasmic reticulum membranes. When ASIC1a is stimulated, it can be activated and transported to the cell membrane. The opening of the channel allows extracellular Ca^2+^ influx, which in turn causes a series of physiological and pathological changes in the cell (Babinski et al., 1999). The common characteristics of these pathological reactions are an acidification of the local tissue environment and a decrease in pH (Kollarik et al., 2007; Gründer and Chen, 2010). Our previous studies have shown that ASIC1a is highly expressed in fibrotic liver tissues in rats and hepatic stellate cells treated with the platelet-derived growth factor PDGF-BB (Pan et al., 2014; Wu et al., 2014). ASIC1a channels promote liver fibrosis by increasing intracellular Ca^2+^ concentrations. This experiment first used Western blotting to verify the expression of ASIC1a and the fibrosis-related proteins α-SMA and Collagen-Ι in HSC-T6 cells treated with CuSO_4_. The results showed that the expression of these proteins increased after copper loading, indicating that CuSO_4_ treatment promoted HSC activation and that ASIC1a plays a role in this process. Psalmotoxin1 (PcTX-1) is a tarantula polypeptide composed of 40 amino acids, and it is a specific blocker of ASIC1a but has no effect on other ASIC channels (Escoubas et al., 2000). Amiloride blocks the inactivation phase of all these channels, but it also blocks other transport systems at relatively high concentrations (Escoubas et al., 2003). After using specific inhibitors PcTX-1 and siRNA to block the expression of ASIC1a, the protein and mRNA expression of the HF marker proteins α-SMA and Collagen-Ι decreased significantly, suggesting that ASIC1a is involved in regulating α-SMA and Collagen-Ι expression. In the liver tissue of copper-treated rats, the expression of ASIC1a, α-SMA, and Collagen-Ι were significantly increased. After gavage with the nonspecific calcium channel inhibitor Amiloride, protein expression was downregulated, which is consistent with the results of the cell model. Interestingly, D-penicillamine, a drug used in WD treatment, also downregulates the expression of α-SMA and Collagen-Ι. It has been reported in the literature that this drug can also be used to treat fibrosis, but the effect is not ideal.

Studies have shown that the activation of ASIC1a induces an influx of extracellular calcium, and disturbances in the ER environment, such as reduced intracavity calcium concentration or changes in redox status, can affect protein folding and processing (Krebs et al., 2015). A reduction in the folding capacity and an accumulation of misfolded proteins in the ER activate a series of signalling pathways, collectively known as the ERS response or UPR. Mitogen-activated protein kinases (MAPKs) belong to the silk protein/threonine kinase family. MAPKs are important molecules that accept signals transmitted by receptors and transduce those signals to the nucleus. It has an important mechanism involved in gene expression regulation, cell proliferation, and death and plays a vital role in various receptor signal transmission pathways (Sun et al., 2015). The P38MAPK signaling pathway is an essential member of the MAPK family and plays a vital role in inflammation and stress response. Recent studies found that the expression of ATP7B mutants activated the P38 MAPK kinase signalling pathway and inhibited the transport of ATP7B from the ER to the Golgi, which was conducive to the rapid degradation of the mutants. Due to the degradation in the ER, the mutant protein is only expressed at 20% of the wild-type protein, so rapid degradation may be the main reason for the loss of ATP7B function in HLD patients. To further explore the mechanism by which ASIC1a is involved in HLD fibrosis, in this experiment, CuSO_4_ was used to establish copper accumulation models in Wistar rats and HSC-T6 cells, and the expression of ERS protein markers and phosphorylated P38 MAPK was detected. The Western blotting and qRT-PCR results showed that the expression of the ERS-related factors GRP78 and XBP1 was significantly increased after treatment with high copper levels, and phosphorylation of the P38 MAPK was increased. However, after gavaging rats with amiloride or pretreating HSC-T6 cells with PcTX-1 or ASIC1a-siRNA, the expression of the abovementioned proteins was reduced. It is suggested that ASIC1a participates in the occurrence of ERS through the P38MAPK pathway and regulating the expression of ASIC1a can attenuate the ERS, which can reduce the degradation of unfolded and misfolded proteins, thus providing a possibility for the retention of ATP7B mutants.

**FIGURE 9:**
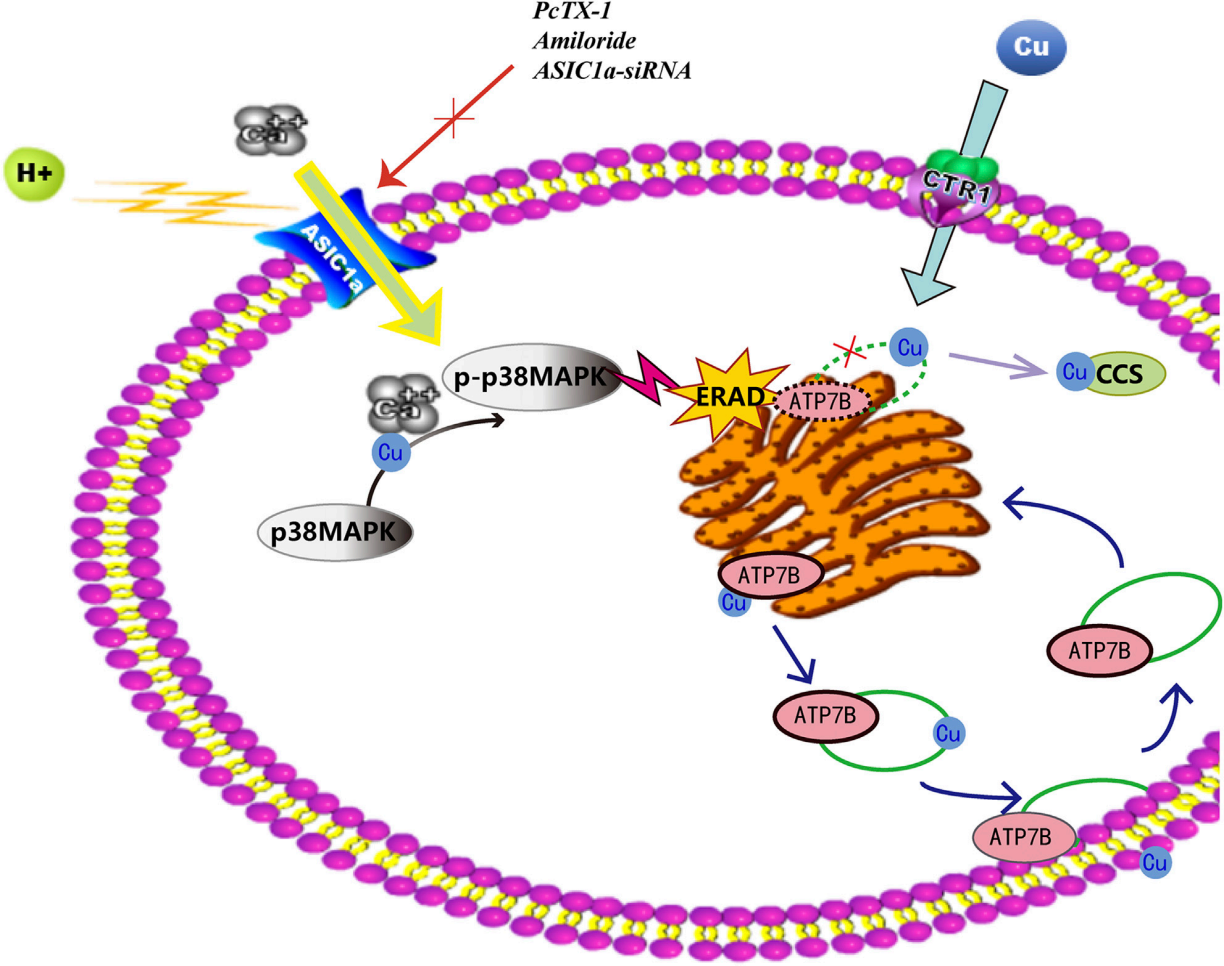
Endoplasmic reticulum stress mediated by ASIC1a through the P38 MAPK signalling pathway contributes to the activation of HSCs and the degradation of ATP7B induced by copper.

In summary, as shown in Figure 9 that ASIC1a expression increases in HF tissues of copper-loaded rats and in HSC-T6 cells treated with CuSO_4_. ASIC1a is activated and opened, and the influx of extracellular Ca^2+^ induces the UPR in HSCs and accelerates ATP7B mutant degradation in the ER. During ATP7B mutant degradation, activation of the P38 MAPK signalling pathway regulates ERS and ASIC1a expression through a positive feedback loop. Rescuing the ATP7B mutant in the ER may be an attractive approach for treating HLD. However, the specific mechanisms by which ERS is activated and stimulation signals are delivered to ASIC1a are not yet clear. The extent to which the inhibition of these pathways can prevent the toxic accumulation of copper in model animals or patients with corresponding ATP7B mutations remains to be determined.

## Data Availability

The raw data supporting the conclusions of this article will be made available by the authors, without undue reservation.

## Abbreviations

ALT, alanine aminotransferase; ASICs, acid-sensitive ion channels; ASIC1a, acid-sensitive ion channel 1a; AST, aspartate aminotransferase; Collagen-I, collagen type I; ECM, extracellμLarmatrix; ER, endoplasmic reticulum; ERS, endoplasmic reticulum stress; GRP78, glucose-regulated protein78; HE, hematoxylin-eosin; HF, hepatic fibrosis; HLD, hepatolenticular degeneration; HSCs, hepatic stellate cells; IL, interleukin; MAPK, mitogen-activated protein kinase; PcTX-1, psalmotoxin-1; TNF-α, tumor necrosis factor-α; UPR, unfolded protein response; XBP1, x-box binding protein; α-SMA, α-smooth muscle actin.
